# Real-Time Belt Deviation Detection Method Based on Depth Edge Feature and Gradient Constraint

**DOI:** 10.3390/s23198208

**Published:** 2023-09-30

**Authors:** Xinchao Xu, Hanguang Zhao, Xiaotian Fu, Mingyue Liu, Haolei Qiao, Youqing Ma

**Affiliations:** 1 School of Geomatics, Liaoning Technical University, Fuxin 123000, China; 472120786@stu.lntu.edu.cn (H.Z.); 472120798@stu.lntu.edu.cn (X.F.); lmy19971109@126.com (M.L.); 18941849570@163.com (H.Q.); mayq@radi.ac.cn (Y.M.); 2 Aerospace Information Research Institute, Chinese Academy of Sciences, Beijing 100101, China

**Keywords:** real-time belt deviation detection, depth edge features, deep learning, gradient constraints

## Abstract

Aiming at the problems of the poor recognition effect and low recognition rate of the existing methods in the process of belt deviation detection, this paper proposes a real-time belt deviation detection method. Firstly, ResNet18 combined with the attention mechanism module is used as a feature extraction network to enhance the features in the belt edge region and suppress the features in other regions. Then, the extracted features are used to predict the approximate locations of the belt edges using a classifier based on the contextual information on the fully connected layer. Next, the improved gradient equation is used as a structural loss in the model training stage to make the model prediction value closer to the target value. Then, the authors of this paper use the least squares method to fit the set of detected belt edge line points to obtain the accurate belt edge straight line. Finally, the deviation threshold is set according to the requirements of the safety production code, and the fitting results are compared with the threshold to achieve the belt deviation detection. Comparisons are made with four other methods: ultrafast structure-aware deep lane detection, end-to-end wireframe parsing, LSD, and the Hough transform. The results show that the proposed method is the fastest at 41 frames/sec; the accuracy is improved by 0.4%, 13.9%, 45.9%, and 78.8% compared to the other four methods; and the F1-score index is improved by 0.3%, 10.2%, 32.6%, and 72%, respectively, which meets the requirements of practical engineering applications. The proposed method can be used for intelligent monitoring and control in coal mines, logistics and transport industries, and other scenarios requiring belt transport.

## 1. Introduction

Under intelligent work safety development, intelligent and safe production in coal mines is becoming increasingly important [1,2]. Belt safety plays a significant role as the main means of production transport, and belt deviation is the primary safety hazard. Belt deviation poses a hidden production danger, leading to economic losses from material scattering and safety accidents, causing casualties. Therefore, detecting belt deviation is crucial for safety. Traditional belt deviation detection relies on human surveillance, which is labor-intensive and becomes less accurate over time. This poses a major safety risk. However, with the widespread use of sensors, sensor-based belt deviation detection methods are gradually replacing human detection. Compared to human monitoring, sensor-based detection offers advantages, such as high accuracy, saving manpower, and improved detection precision. Currently, image processing-based belt deviation detection methods are being utilized due to the maturity of computer technology. It offers benefits, such as fast detection speed and low cost. However, its accuracy is still relatively low and does not meet intelligence and production safety requirements. Hence, it is of great importance to study a fast and accurate belt deviation detection method that can enhance both efficiency and safety.

At the present stage, there are mainly two types of detection methods, such as contact (sensor data-assisted method) and non-contact (image processing-based method), for the belt deviation detection problem. The contact belt deviation detection method is to install the sensor around the belt facility, trigger the belt deviation signal by detecting the abnormal deviation of the belt, feedback the signal to the terminal, and then complete the belt deviation detection. Fu et al. [3] proposed a temperature-compensated ultrasonic rangefinder for belt deviation detection, which completes the belt deviation detection by calculating the distance value between the sensor and the belt in comparison with the set distance value threshold. Wei et al. [4] proposed distributed fiber-based detection equipment, which collects real-time data during the operation of the belt’s mechanical structure and analyzes and feeds back to find out whether there is a belt deviation phenomenon or not. Wang Xu et al. [5] proposed three-dimensional synchronized belt deviation detection, which detects the belt deviation through real-time three-dimensional modeling by LiDAR. Zeng et al. [6] designed a belt deviation detection system based on laser scanning. The method uses a laser scanner to obtain the point cloud data of the conveyor belt in real time, performs data processing to obtain the edge information of the belt, and utilizes the information obtained from the speed correction model constraints to fit the image. The geometric information of the image is used to determine the belt deflection. Sensor-assisted detection methods are more accurate, but this type of method has a higher cost and poor environmental adaptability, maintenance and repair is more difficult, the detection results still need to be combined with the relevant software reasoning, resulting in the universality of application scenarios being limited, and the detection speed is slow. With the maturity of image processing technology, many scholars use it for belt deflection detection, that is, a non-contact belt detection method based on image processing.

An image processing-based belt deviation detection method is mainly based on image features and deep learning means to detect the belt deviation behavior. Bi et al. recognized the belt edge by Canny edge detection based on video images and judged the running status of the belt by comparing the edge line with the threshold line to accomplish the detection task [7,8,9]. Liu et al. [10] proposed a method for detecting the deviation from an arbitrary position of the belt conveyor based on inspection robots and deep learning, which extracts the edge of the belt by using the Hough transform to offset the estimation to realize the belt deviation detection. Zhang et al. [11] proposed a conveyor belt deviation monitoring method with edge detection, which uses edge detection to detect the edge of the conveyor belt and determine the angle between the edge line and the positive direction of the belt operation to quickly determine whether the belt is deviated or not. Sun et al. [12] proposed a combined ARIMA-LSTM-based belt deviation state assessment system, which uses a trained model for real-time belt deviation state detection, prediction, deviation correction, and warning. Although the above methods can theoretically achieve the accurate detection of belt deviation, none of them are applied in practical scenarios and do not have visualized belt detection effect diagrams. Zeng et al. [13] used a multi-scale feature fusion network structure to fuse low-level features with information such as the position, extracted the belt features by using a depth-separable network, and computed the belt deviation in order to achieve belt detection. However, the real-time performance of this method is poor. Zhang et al. [14] increased the ability of the network to detect straight lines by improving the output of yolov5 [15], which in turn accomplishes the fast extraction of belt features and deviation analysis. The belt detection method based on image features has good results in both detection accuracy and detection speed, but it is greatly affected by the imaging environment, such as image noise and low-texture scenarios caused by excessive dust and weak lighting conditions, which will reduce the detection rate of the belt features. Therefore, in order to avoid the redundancy of image features and improve the robustness of belt detection, many scholars have carried out a great deal of research work on the problem of line feature extraction, taking into account the nature of belt features, i.e., line features. The existing line feature detection methods are roughly categorized into edge connection-based methods, image gradient-based methods, and deep learning-based methods.

A representative of the edge connection-based line detection algorithms is Hough line detection. Duda et al. [16] proposed the Hough line detection method. This method utilizes the duality of points and lines, which means that the co-linear points can be mapped as intersecting straight lines under the polar coordinate system, and their intersection points are the straight lines under the image coordinate system. The intersection points in the parameter space are counted and voted to achieve straight line detection in the image. Lu et al. [17] proposed an adaptive parameter Canny algorithm for edge feature extraction for Hough straight line detection, which solves the problem of the loss of edge detection features with a set threshold and then optimizes the accuracy of Hough’s line extraction. The results of the above edge connection-based straight line detection algorithm detection effect of a good a priori condition is to have a better edge detection; conditions such as weak light will lead to poor edge detection, directly affecting the effect of straight line detection.

Line detection methods based on image gradient methods detect straight lines in an image with the gradient features of pixels. Von Gioi et al. [18] proposed line segment detection (LSD), an algorithm that determines the seed points based on the image gradient information and performs the region growing method to establish the connectivity domain. The screening and determination of straight lines is carried out through the minimum outer rectangle principle and Helmholtz principle. Cho et al. [19] proposed a method based on the intrinsic properties of straight lines in the line segment slice simulation space. The method suppresses the gradient magnitude by NMS for horizontal or vertical connected pixels for line segment slice detection, clusters the line segment slices according to the extracted image features, and verifies the straight line segments by using clues such as the gradient magnitude and gradient direction to determine whether the detection results are correct or not. Liu et al. [20] proposed a luminance-weighted sub-interval straight line extraction method. This method models the curve adaptive division with the gradient direction using Gaussian distribution and determines the threshold value in each gradient direction to complete the initial straight line extraction using the luminance of the pixel as the weighting dependency. Then, straight line joining is performed with center of gravity consistency and endpoint distance constraints. Moon [21] a P&ID line object extraction method, which uses the edge detection of straight lines with differential filters in the algorithm. The abovementioned image gradient-based straight line detection methods have good detection results, but the detection speed is slow.

Deep learning-based line feature detection methods have been widely used in wireframe detection, semantic line detection, and lane line detection, which are the main line feature detection methods nowadays. Zhou et al. [22] proposed an end-to-end linear detection method (L-CNN). The method predicts candidate straight line connecting points by taking the extracted image features at the pixel level, predicts straight lines based on the candidate points, uses NMS suppression, and completes the straight line detection. Zhang et al. [23] proposed a straight line detection method with a graph approach. The method first identifies all the connecting points and then calculates the connectivity of the points as the confidence level of the line segment information. Xu et al. [24] proposed transformer-based line segment detection. The method uses two prediction heads on top of the transform decoder for line segment prediction. Zhao et al. [25] proposed a semantic line detection method. The method combines the feature extraction capability of a CNN with a Hough transform to realize a real-time accurate semantic line detection method. LineCNN [26], LaneATT [27], and other methods have been designed with a method based on the line anchor, which uses a linear relationship between the sampling points and the preset offsets and regresses to derive the lane lines with the highest level of confidence using NMS as a constraint. The above methods have an excellent performance ability in most of the scenes, and the scene distribution is mostly based on sufficient illumination and rich image contrast. The performance ability for scenes with weak illumination, a simple texture, and low contrast will be reduced, resulting in lower detection accuracy.

In general, the above methods have made some degree of progress in the field of belt deviation detection methods and line feature extraction. However, they cannot satisfy the universality of the scene of the application field, nor can they guarantee the detection accuracy with a real-time detection speed at the same time. This paper proposes a real-time belt deviation detection method based on depth edge features and gradient constraints (RBDNet). We summarize our contributions as follows:

(1) Initial belt edge line position estimation: This method utilizes contextual information and belt edge features to estimate the position of the belt edge line. This estimation is achieved by dividing the image from the line anchor [28] within the belt region.
(2) Constraint using the first-order difference method: This method employs a first-order difference approach to constrain the belt edge features, ensuring a fast and accurate belt edge line determination.
(3) This method involves constructing an industrial belt deviation detection dataset with multiple scenarios and angles from the collected field monitoring data.
(4) In abnormal situations, this paper still shows a more accurate detection effect, such as in the case of the belt area shelter and excessive dust.
(5) Advantages over conventional algorithms: Compared to traditional straight line detection algorithms, the proposed method excels in extracting belt edge straight lines with high accuracy and speed, avoiding false detection and missed detection. Advantages over sensor-based methods: In comparison to sensor-based belt deviation detection methods, RBDNet offers lower environmental adaptability requirements, reduced cost and maintenance, and improved detection accuracy. It performs better in harsh and complex environments, such as downhole scenarios.
(6) Improvements over existing image processing-based methods: This method addresses the limitations of time-consuming detection and low detection accuracy that commonly exist in current image processing-based belt deviation detection methods. The visualization of the belt edge lines and threshold lines in the detection results enhances the recognition and effectively solves the issues of slow detection speed and low accuracy seen in previous methods.

Overall, the proposed RBDNet method provides a real-time belt deviation detection solution that combines depth edge features and gradient constraints, offering higher accuracy, a faster detection speed, and improved visual representation compared to the existing approaches.

## 2. Materials and Methods

**Figure 1:**
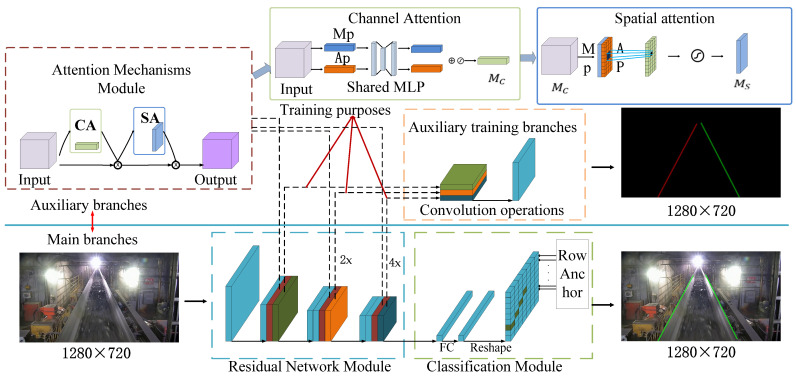
Overall structure diagram. In Figure 1, CA is channel attention mechanism, SA is spatial attention mechanism, MP is max pooling, AP is average pooling.

The proposed method utilizes the residual network (ResNet) as the backbone network model. It focuses on retrieving edge features that are specifically related to the straight lines along both sides of the belt. By doing so, the model is optimized for speed and efficiency, leading to an improved inference speed in belt deviation detection. Once the edge features are obtained, the algorithm employs least squares linear fitting to accurately estimate the belt edge straight lines. These lines are then compared with a predefined deviation threshold to determine whether there is any belt deviation. Figure 1 illustrates the overall algorithm architecture, showcasing the flow and organization of its various components. In summary, the method relies on ResNet as the backbone model, retrieves the edge features relevant to the belt edge lines, optimizes the model for speed, employs least squares linear fitting for accurate estimation, and compares the detected lines with a deviation threshold to detect belt deviation. This paper provides a detailed depiction of the algorithm’s architecture in Figure 1.

### 2.1. Network Feature Propagation and Model Construction

The residual network (ResNet) [29] has a deeper network structure compared to other networks. The use of shortcut connection blocks enables deeper network layers, preventing overfitting issues that may arise from excessively deep layers. ResNet models include variants, such as ResNet18, ResNet34, and ResNet50. Generally, increasing the number of layers improves the model prediction accuracy but also increases the parameter count and reduces the inference speed. In this paper, ResNet is utilized as the backbone network, with the ParsingNet module [30] employed during training to refine the shape and position of both edges of the belt. This module helps improve the accuracy of belt edge detection by further adjusting the network’s perception of the belt features. To summarize, the authors of this paper take advantage of ResNet’s ability to handle deep networks, utilizing shortcut connections. The chosen ResNet variant serves as the backbone network, while the ParsingNet module contributes to precisely refining the belt edge shape and position during training.

The method described in this paper utilizes a convolutional neural network (CNN) with four key modules: the input–output layer, convolutional layer, pooling layer, and fully connected layer. Here is an overview of the method’s architecture: (1) Input–Output Layer: The belt surveillance video images are standardized through the data input layer to ensure consistent formatting. (2) Convolutional Layer: The standardized data are fed into the convolutional layer for feature extraction. This layer applies convolutional operations to capture the relevant features from the input data. (3) Pooling Layer: The output results from the convolutional layer are standardized and then passed through the pooling layer using the rectified linear unit (ReLU) activation function. The average pooling (MaxPool) and maximum pooling (AvgPool) operations are performed on the extracted features to enhance their information. (4) Fully Connected Layer: The image features obtained are organized in a distributed manner in the fully connected layer. The learned distributed feature representation is mapped to the sample’s marker space. The cost calculation of the features is conducted to accurately detect the exact location of the belt edges. The detection results are visualized and displayed through the output layer. In summary, this method employs a CNN architecture with standardized inputs, feature extraction through convolutional and pooling layers, distribution-based organization of the features in the fully connected layer, and precise edge detection through cost calculation. The detection results are then presented via the output layer.

### 2.2. Belt Edge Attention Mechanism

The method described in this paper enhances the performance of focusing on belt edge feature information and suppressing unnecessary features in scenarios such as coal transport belts where factors such as lighting locations and dust can affect the lighting conditions. It achieves this by designing and applying a transformer mechanism in the mod module [31,32].

The input belt feature *F* is passed through the parallel *MaxPool* and *AvgPool* layers, changing the feature size from C × H × W to C × 1 × 1, where C is the number of image channels, H is the image height, and W is the image width. The shared *MLP* module first compresses the number of feature channels to 1/r (r is the reduction rate) times the original number of channels and then reduces it to the original number of channels. The *MaxPool* and *AvgPool* layers are activated by the ReLU activation function to obtain the results. The two output results are summed element by element, and then the output result of the channel attention *M_c_* is obtained by the sigmoid activation function. *M_c_* is then multiplied by the element with the corresponding input features and restored to the dimension of C × H × W. The new belt feature, *M_c_(F)*, after the channel attention mechanism operation, is shown in Equation (1) below.

(1) $$M_{c}\left(F\right)=\sigma (MLP\left(AugPool\left(F^{c}\right)\right)+MLP\;\left(MaxPool\left(F^{s}\right)\right))$$

*M_c_(F)* is passed through the *MaxPool* and *AvgPool* operations to obtain the corresponding two 1 × H × W belt features. The belt features of the two different operations are concatenated by the Concat (channel concatenation) operation, and the belt features are dimensionally reduced by the 7 × 7 convolution to transform the concatenated features into belt features with one channel. The new features *M_s_* are then obtained by sigmoid activation. Finally, *M_s_* is multiplied with the corresponding input features by the element to recover the C × H × W dimension. The final belt feature *M_s_(F)* after the spatial attention mechanism operation is shown in Equation (2).

(2) $$M_{s}\left(F\right)=\sigma (f^{7\times 7}\left[AugPool\left(F^{s}\right);MaxPool\;\left(F^{s}\right)\right])$$

The final features *M_c_(F)* and *M_s_(F)* after the channel attention and spatial attention processing are arranged serially to obtain the final feature descriptions as input to the fully connected layer.

### 2.3. Position Prediction Based on Belt Edge Features

In this paper, the belt edge line position is predicted accurately from top to bottom in different scenes and viewpoints. The approach for position prediction depends on the availability of the belt edge features. 

#### 2.3.1. Complete Belt Edge Features

In scenes where the belt edge features are complete, the prediction is carried out using these features. These features provide sufficient information about the belt edge position, allowing accurate prediction. 

#### 2.3.2. Partially Missing Belt Edge Features

In scenes where there are belt edge features that are partially missing or obscured, global features are used for position prediction. Global features capture the overall information about the scene and do not rely solely on the belt edge. This helps to predict the position even when some belt edge features are unavailable or obscured. By utilizing both the belt edge features and global features, the position prediction method adapts to different scenarios and ensures accurate predictions regardless of the completeness of the belt edge information in the scene.

**Figure 2:**
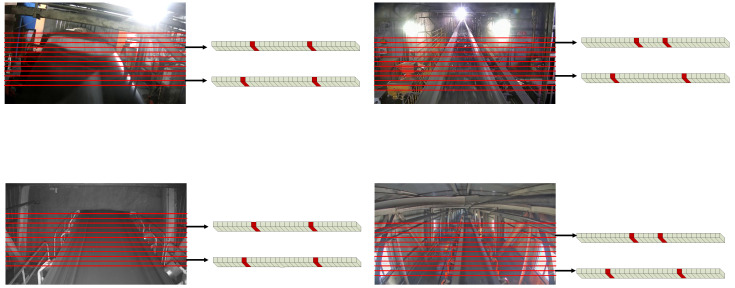
Division of row anchors and determination of the belt edge lines position. The red lines in the figure show the division of the row anchors. The yellow squares are the cells divided according to the row anchors and the red squares are the cells where the belt edges are located. (Note: In Figure 2, the image height of the dataset in this paper is 720. According to the actual situation, this paper sets the range of row anchors: Scene 1, from 140 to 690 with a step of 10; Scene 2, from 160 to 710 with a step of 10; Scene 3, from 140 to 690 with a step of 10; Scene 4, from 150 to 700 with a step of 10.)

The belt edge location prediction requires a line anchor for the area of the image where the belt is located. The anchors are selected from the main area of the image where the belt is located, i.e., the area where the belt edge features are relatively prominent, up to 70% of the image height. Scientific and reasonable row anchor delineation can help to predict the belt edge location more accurately, reduce the number of computational parameters, and reduce model complexity. The row anchor delineation in different scene images varies according to the position of the belt in the image, as shown in Figure 2.

To improve the operating efficiency, after the row anchor division is completed, it is necessary to grid each row anchor into multiple cells where the edge position prediction is performed. Assuming that the grid point is the probability of the belt, *P_i,j:_* is a *w +* 1 dimensional vector, indicating the probability of the *i*th belt edge line, on the *j*th row anchor, on the *w + 1*st grid cell, and then its calculation formula is as follows:

(3) $$P_{i,j,:}=f^{ij}\left(X\right),i\in \left[1,C\right],j\in [1,h]$$

where *C* is the maximum number of belt edge lines, *h* is the number of row anchors, *X* is the global feature after pooling, and w is the number of gridded cells. *f^ij^* is used to select the classifier on the *i*th belt edge line, on the *j*th row anchor, for predicting where the belt edge line might be located. To improve the probabilistic prediction accuracy, this paper uses cross-entropy loss *L_CE_* in *w +* 1 dimensions for the loss of the belt edge lines. Assuming that *M_i,j_* is the label of the correct position with the highest probability, the prediction accuracy of the edge position *L_cls_* can be obtained by combining *P_i,j:_* with Formula (4) as

(4) $$L_{cls}=\sum_{i=1}^{c}\sum_{j=1}^{h}L_{CE}(P_{i,j,:},M_{i,j,:})$$

As the probability of the belt edge position is predicted according to continuous linear features, the belt edge feature disappears in the presence of occlusion, and no direct position prediction can be made. Other methods result in false detections or false deviation warnings in this case. In this paper, masking is divided into two cases: one belt edge is masked (one-sided masking), and both belt edges are masked (bilateral masking).

Single-sided masking: First, the position of the unmasked edge on the other side is calculated by Equation (3) and combined with the continuity characteristics of the belt edge on the adjacent row anchor. Then, the probability of the belt edge position on the masked side *P_i-_*_1*,j:*_ can be obtained as

(5) $$P_{i-l,j,:}=f^{i-lj}\left(X\right),i-l\in \left[1,C\right],j\in [1,h]$$

where *l* is the belt width obtained in advance in the video image when unmasked.

Bilateral masking: When both sides are masked, the positions of the two belt edges on the currently masked row anchors cannot be obtained and need to be predicted by the continuity characteristics of the belt edges on the adjacent row anchors. Assuming that the probability of a belt edge obtained by belt edge prediction within a grid point at the location of one edge at the proximal breakpoint is *P_i_*_1*,*1*j:*_, the probability of a belt edge obtained by belt edge prediction within a grid point at the distal point location is *P_i_*_2*,*2*j:*_. To determine the belt edge location within the occlusion region, the contextual information of the belt edge needs to be known in advance. The maximum of *P_i_*_1*,*1*j:*_ and *P_i_*_2*,*2*j:*_ is taken as the most likely belt edge location at the endpoints *Loc1* and *Loc2*:

(6) $$Locg=\arg\max P_{ig,jg.:},g=1\;,\;2$$

Belt position prediction in the occlusion scenario is achieved by considering contextual information and using global features. The candidate points for the belt edge in the masked region are distributed near the line connecting *Loc1* and *Loc2*. By utilizing the belt edge continuity and the probability distribution of known locations, the probability of the belt edge points’ location in the occluded region can be reduced. The final prediction accounts for belt edge continuity in the line anchor and the geometry constraint of the belt width.

### 2.4. Belt Edge Losses

This paper utilizes two loss functions to determine the mixed position relationship of the belt edge line points. The contextual information of the global features is employed in calculating both the local similarity loss and the belt edge shape loss. These loss calculations help constrain the belt edges and determine their positions. Subsequently, a feature aggregation method is applied to generate a set of candidate points for the belt edge.

#### 2.4.1. Similarity Loss

Similarity loss is mainly determined by a combination of continuity and similarity. The belt edge features are calculated within the row anchor region, which is continuous, thus guaranteeing the continuity condition for the belt edge feature continuity loss calculation. The similarity of the edge features is guaranteed because the similar row anchor area is divided by the entire belt, which has typical linear characteristics. The belt position probability obtained by using the feature variation, combined with the adjacent row anchor’s predicted belt edge probability, enables the similarity loss cost calculation by calculating the *L1* parametrization of both. The similarity loss is defined as

(7) $$L_{sim}=\sum_{i=1}^{c}\sum_{j=1}^{h}\left\|P_{i,j-1,:}-{\left.P_{i,j,:}\right\|}_{1}\right.$$

where ${\left\|\cdot \right\|}_{1}$ denotes the *L1* parametrization.

#### 2.4.2. Shape Loss

The belt edge line can be approximated as a straight line, so the consistency of the first-order difference equation can be used as a constraint on the shape of the belt edge line. When the shape is linear, the result of the first-order difference equation for the belt edge feature should be constant, i.e., the slope of the line at the edge remains constant, whereas if the difference equation is not constant, the feature is not consistent with the nature of the belt edge and can be discarded. In this paper, the belt edge feature is determined using a first-order difference equation, and all the functions within the equation should satisfy the condition of being differentiable, so the probabilities of different location image features are obtained using the differentiable softmax function, i.e., the predicted expectation as an approximation of the location feature, as shown in Equation (8):

(8) $${Prob}_{i,j,k}=\frac{exp{(P}_{i,j,k})}{\sum_{z=1}^{w}exp{(P}_{i,j,k)}},\;(k=1,2,3,\ldots ,w)$$

The expectation of the location of the belt edge can be expressed as

(9) $${Loc}_{i,j}=\sum_{k=1}^{w}{kProb}_{i,j,k}$$

where *Prob_i,j,k_* denotes the probability of the belt edge being the location of falling on the *i*th belt edge line, the *j*th row anchor, and the *k*th unitized grid. The expectation of a discrete random variable from a probability distribution is used to determine the maximum expectation, i.e., the exact position of the belt edge line. In summary, the shape loss is defined as

(10) $$L_{shp}=\sum_{i=1}^{c}\sum_{j=1}^{h}\left\|{(Loc}_{i,j}-{\left.{Loc}_{i,j-1})\right\|}_{1}\right.$$

Ultimately, the combination of the belt edge position prediction, similarity loss, and shape loss cost calculation gives a final loss *L_total_* of

(11) $$L_{total}=L_{cls}+\alpha (L_{sim}+{\beta L}_{shp})$$

where α and β are the loss factors.

Based on the above constraints, the belt edge locations are determined in conjunction with the belt edge location predictions in Section 2.3. The belt edge features are then aggregated and modeled pixel by pixel to obtain the belt edge candidate points.

### 2.5. Linear Fitting of the Belt Edge Point Set

The set of the identified belt edge candidate points is arranged in the direction of the belt edge line and the straight line fit is performed to the set of points to generate the belt edge line to be detected. Before performing the straight line fit, the deviation between the actual and predicted values needs to be estimated based on the pixel positions, and then a suitable fitting method needs to be determined, as follows:

1. A function *f(x;*$a_{1},a_{2},a_{3},\ldots ,a_{n}$*)* and its pixel values at *N* different belt edge candidates *(x*_1_*,y*_1_*), (x*_2_*,y*_2_*), (x*_3_*,y*_3_*),…(x_n_, y_n_)*, i.e., *f(x;*$a_{1},a_{2},a_{3},\ldots ,a_{n}$*)*, is given.
2. Determine the n-term coefficients *a*_1_*,a*_2_*,a*_3_*…,a_n_* of the unknown parameter set to derive the formula for the deviation *r_i_* of candidate point *i*:
  (12) $$r_{i}=f\left(x_{i};a_{1},a_{2},a_{3},\ldots ,a_{n}\right)-y_{i},\;i=1,2,3,\ldots N$$
3. The number of candidate points *N* should be greater than the number of parameters *n* to reduce the influence of invariant errors in the input data. Assuming that the constant variance in the vertical coordinate *y_i_* of the candidate pixels satisfies a normal distribution, the Gaussian fitting principle or the least squares method is applicable according to probability theory. With minimum deviation, the following equations can be determined for the solution of each coefficient:
  (13) $$\sum_{i=1}^{N}r_{i}^{2}=\sum_{i=1}^{N}[f\left(x_{i};a_{1},a_{2},a_{3},\ldots ,a_{n}\right)-y_{i}{]}^{2}$$
4. In this paper, we choose to fit the belt edge points by the least squares fitting method, assuming that the equation of the belt edge line is *y = ax + b*, and the fitting Formula (14) is as follows:
  (14) $$\left\{\begin{array}{c} \hat{b}=\frac{\sum_{i=1}^{n}\left(X_{i}-\bar{X}\right)\left(y_{i}-\bar{y}\right)}{\sum_{i=1}^{n}{\left(X_{i}-\bar{X}\right)}^{2}}=\frac{\sum_{i=1}^{n}x_{i}Y_{i}-n\bar{X\bar{y}}}{\sum_{i=1}^{n}x_{i}^{2}-n\bar{X^{2}}}\\ \hat{a}=\bar{y}-\hat{b}\bar{x} \end{array}\right.$$
  where $\hat{a}$ and $\hat{b}$ are the least squares to obtain the linear coefficient valuations and $\left(\bar{x},\bar{y}\right)$ are the mean values of the coordinates of the set of candidate points. According to the above formula, substitute all the belt edge candidate points, carry out linear fitting, construct the linear equation of the belt edge line, visualize the belt edge detection results, attain the belt edge, and compare it with the set deviation threshold. If it exceeds or is compared to the threshold line, it is judged as the belt deviation; perform alarm processing, and stop the belt machine operation through the signal control device.

### 2.6. Setting the Belt Deviation Threshold Line

When the belt conveyor is in normal operation, the belt is in a closed loop, the upper and lower belts run at constant speed, and the center lines run parallel to each other. The offset in the belt width direction is to be kept within the specified limits. In accordance with the safety standards for belt machines, a belt with a deflection of more than 5% of the belt width is considered to have deviation. In accordance with the above conditions, the deviation threshold line is defined in this paper by the following equation:

(15) $$D_{s}=\frac{D_{ST}W_{B}}{{Pix}_{xL}-{Pix}_{yR}}$$

where *D_S_* is the maximum belt offset in the image, *D_ST_* is the actual maximum belt offset, *W_B_* is the actual width of the belt, *Pix_xL_* is the pixel x-coordinate of the left edge of the belt in the image, and *Pix_xL_* is the pixel x-coordinate of the right edge of the belt in the image. From this, the maximum offset of the belt in the image can be derived. The belt deviation threshold line is based on the pixel x-coordinate of the belt edge line plus (minus) the maximum offset when the belt is in normal operation (and in the middle of the carrier rollers), which is the pixel x-coordinate of the belt deviation threshold line. Based on the point-slope equation for a straight line, the belt deviation threshold line is obtained using the top left and bottom left, and the top right and bottom right points of the belt in the image are a basis. The left belt edge line Equation (16) shows that

(16) $$y_{thL}=\frac{y_{LU}-y_{LD}}{x_{LU}-x_{LD}-D_{s}}x_{thL}+(y_{LU}\frac{y_{LU}-y_{LD}}{x_{LU}-x_{LD}-D_{s}}-x_{LU})$$

where (*y_thL_*,*y_thL_*) is the set of points of the left edge line, (*x_LU_*, *y_LU_*) are the pixel coordinates of the upper left point of the belt, and (*x_LD_*, *y_LD_*) are the pixel coordinates of the lower left point of the belt.

The right belt edge line Equation (17) shows that

(17) $$y_{thR}=\frac{y_{RU}-y_{RD}}{x_{RU}-x_{RD}-D_{s}}x_{thR}+(y_{RU}\frac{y_{RU}-y_{RD}}{x_{RU}-x_{RD}-D_{s}}-x_{RU})$$

where (*y_thR_*, *y_thR_*) is the set of points of the left edge line, (*x_RU_*, *y_RU_*) are the upper right point pixel coordinates of the belt, and (*x_RD_*, *y_RD_*) are the lower right point pixel coordinates of the belt.

## 3. Results

The algorithm in this paper was first compared with ultrafast structure-aware deep lane detection (UFSLD) [30], L-CNN [22], LSD [18], and the Hough transform [16], where 60% of the image data in the dataset were used to train the model, 20% were used for testing, and 20% were used for validation; the dataset constructed in this paper was used for training and testing. ResNet with different backbone networks was used for the accuracy and speed comparison experiments to determine the optimal backbone network in terms of the accuracy and speed. In addition, to verify the effectiveness of the proposed strategy, ablation experiments were conducted to quantitatively analyze the improved loss function, the added attention mechanism, the improved probability distribution of the belt edge locations, and the straight line fitting of the belt edge candidates to verify the practical effectiveness of the proposed method. A quantitative comparison analysis of the accuracy and speed was carried out to verify the difference in the accuracy and speed between the algorithm in this paper and the other algorithms. According to the actual situation, the proposed algorithm and the other algorithms were carried out in two different scenes for belt edge straight line extraction, and the experimental results were visualized. In addition, masking experiments were carried out to simulate the complexities that may occur during belt transportation using black masks to verify the stability of the proposed method.

### 3.1. Experimental Equipment and Training Parameters

In this paper, the model was trained by combining a self-labeled dataset with the TuSimple dataset. Due to the relatively small size of the experimental dataset compared to the Tucson dataset and the less diverse scene data, a specific dataset suitable for mining belts was created. This dataset underwent data augmentation techniques, including flipping, rotation, random color adjustments, contrast enhancement, and brightness adjustment. These techniques were applied to overcome adverse conditions such as varying lighting and to adapt the model to the complex environments encountered in mining areas.

**Figure 3:**
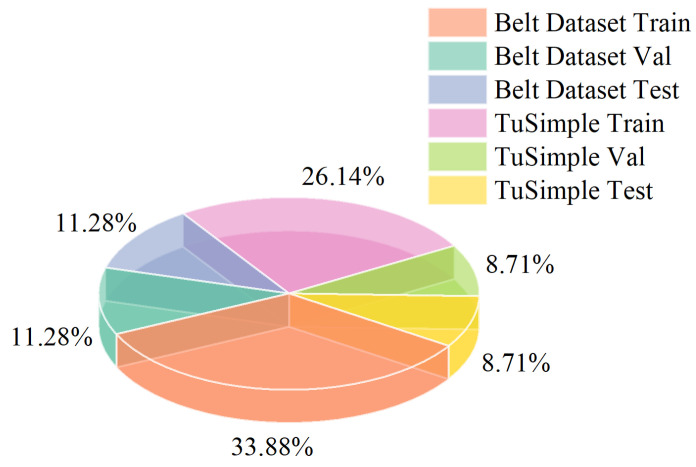
Composition of the dataset.

Mining belt conveyors are typically long, requiring monitoring of multiple sections to assess their real-time operational status. To acquire on-site data, the video data from cameras positioned at different angles were used as a reference. The monitoring videos were then processed frame by frame, and the extracted belt images were manually annotated using “LabelMe” software to create labeled belt edge lines. The real images and the corresponding labeled data and binary images with labels were compiled into a cohesive dataset. Finally, the data augmentation techniques were applied to further enhance the dataset. Figure 3 provides the details of the composition of the dataset.

The model in this paper was trained using the Adam [33] optimizer with a cosine decay learning rate strategy [34] initialized with 0.0004, with the batch processing size set to 16 and the total number of training periods set to 300 for the dataset in this paper. The training parameters for this experiment are shown in Table 1 and Table 2.

### 3.2. Ablation Experiments and Experimental Evaluation

#### 3.2.1. Selection of Backbone Networks

The experiments were conducted using three different backbone networks: ResNet18, ResNet34, and ResNet50. Real-time video streams were used for these experiments, and the performance of each backbone network was evaluated in the detecting surveillance videos. The inference time of each network model was measured, and the evaluation was based on the processing results of one minute of stable operation. The accuracy and F1-measure of the network models using different backbone networks were calculated. The results of these calculations are presented in Table 3.

Based on the information provided in the table, it appears that ResNet18 met the requirement for real-time processing as its inference speed was satisfactory. Additionally, ResNet18 demonstrated a comparable accuracy rate to ResNet34 and ResNet50. However, the inference speeds of ResNet34 and ResNet50 were relatively slower, which may result in blockages in real-time monitoring video streams. Consequently, ResNet18 was selected as the backbone network for practical application.

#### 3.2.2. Ablation Experiments

The effectiveness of RBDNet was verified through three sets of ablation experiments. The results of the quantitative analysis can be found in Table 4. In these experiments, the same platform, virtual environment, and backbone network conditions were maintained. Furthermore, the training parameters remained consistent when different improvement modules were combined to assess their impact.

**Figure 4:**
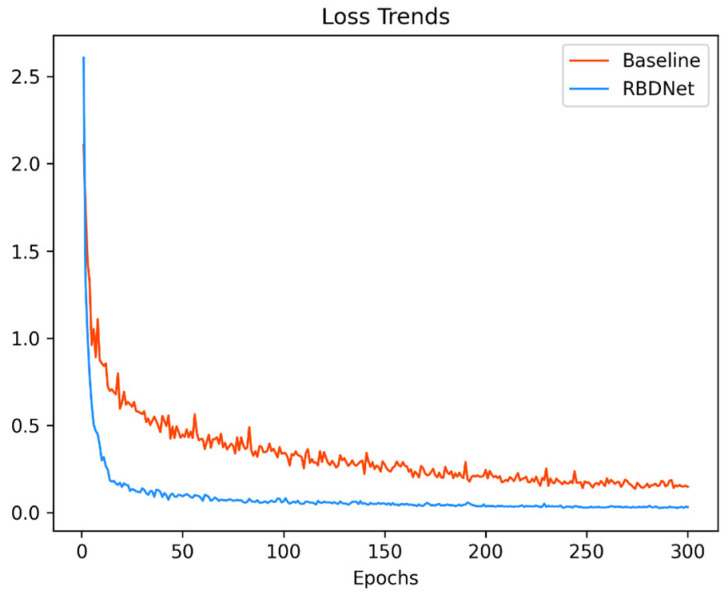
Comparison of loss trends. Figure 4 is the comparison of loss changes during the training process between the baseline model and the REDNet model. In addition, the experimental conditions of the two types of networks are consistent, except for the data source.

Based on the observations in Table 4, it can be deduced that predicting the belt edge position and utilizing the belt edge structure loss with the attention mechanism contribute to enhancing the performance. All three operations exhibited positive effects on the overall accuracy. Notably, the combined utilization of these improvements demonstrated the most substantial enhancement in algorithm accuracy. This outcome further validates the RBDNet effectiveness and reliability. The training loss line graph is shown in Figure 4.

#### 3.2.3. Experimental Evaluation

The accuracy, F1-measure, and inference time of the different algorithms were evaluated for accuracy and speed detection. The accuracy was calculated as follows:

(18) $$accuracy=\frac{\Sigma_{clip}C_{clip}}{\Sigma_{clip}S_{clip}}$$

where *C_clip_* is the number of correctly predicted belt edge points and *S_clip_* is the total number of actual location belt edge points in each image. The cross union (IoU) between the true and predicted belt edge location values was then calculated. Predictions with an IoU greater than 0.5 were considered to be true positives. The evaluation index F1-measure was calculated as follows:

(19) $$F1-measure=\frac{2\times Precision\times Recall}{Precision+Recall}$$

where precision is TP/TP + FP and recall is TP/TP + FN, where TP denotes true positive, FP denotes false positive, and FN denotes false negative. Finally, the accuracy, precision, and running speed of the different methods were determined. The results are shown in Table 5.

**Figure 5:**
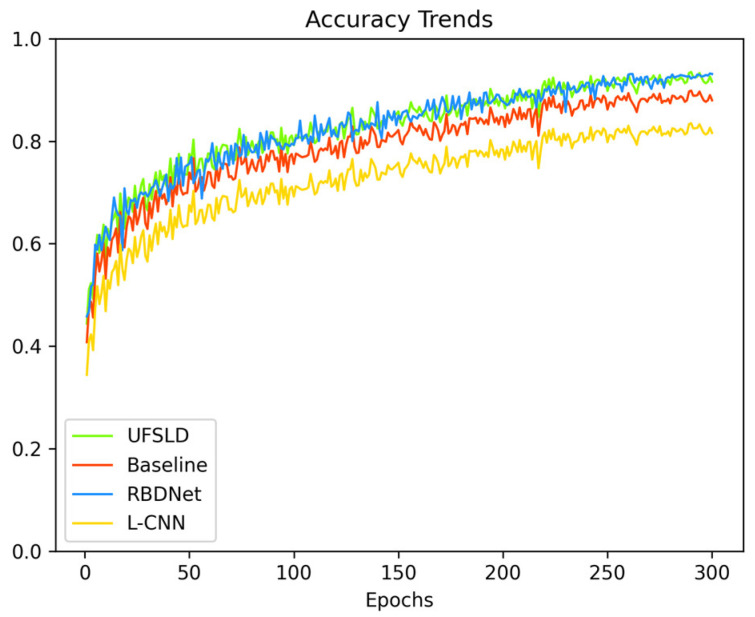
Comparison of accuracy trends. Figure 5 is the comparison of accuracy changes during the training process between the REDnet model, the UFLSD model, and the L-CNN model. In addition, the experimental conditions of the three types of networks are consistent except for the data source.

According to the information provided in Table 5, it is evident that the algorithm presented in this paper achieved the highest accuracy and precision. UFSLD and L-CNN follow closely in terms of performance. In regard to detection speed, both the algorithm in this paper and UFSLD can meet real-time requirements, while L-CNN can meet quasi-real-time requirements. The remaining two methods exhibited relatively poor accuracy and precision, coupled with slower speeds, which limit their practical applicability. The accuracy of different algorithms is shown in Figure 5.

RBDNet utilized belt edge position prediction based on the feature belt edge position to swiftly locate the belt edge position. The belt edge attention mechanism ensures accurate belt edge extraction. Including the first-order difference constraint reduced the program time consumption, ultimately improving the detection speed while maintaining detection accuracy. Consequently, the detection speed of RBDNet reached 41/s.

### 3.3. Comparison of the Detection Effects of Different Methods

**Figure 6:**
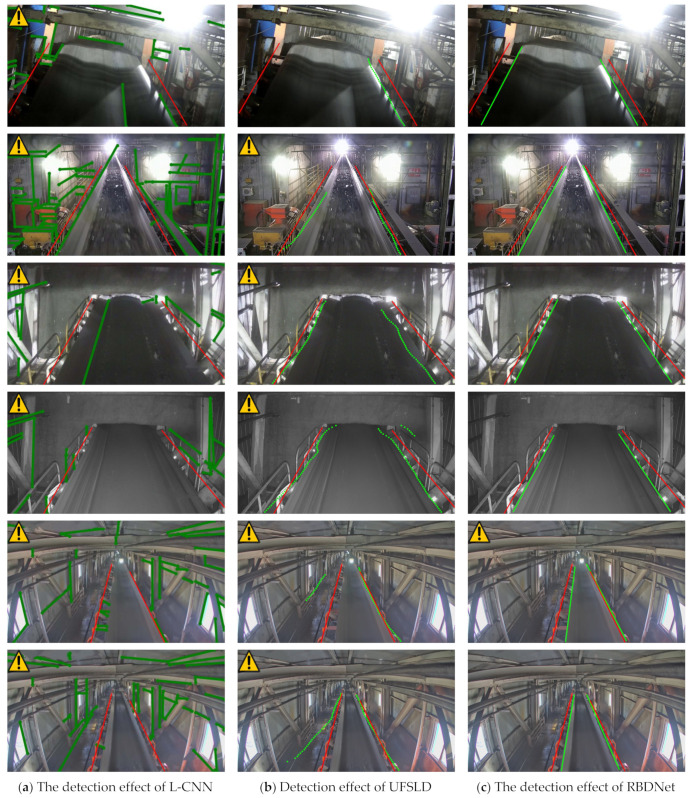
Diagram of the results of belt misalignment detection with different algorithms. In Figure 6, the green line is the detection result and the red line is the runout threshold line.

The algorithm accuracy indices and the UFSLD and L-CNN methods in this paper are not much different from those of RBDNet, but they do not represent their practical application effects. Therefore, the proposed RBDNet was visualized and compared with UFSLD and L-CNN and the other line feature detection methods in different scenarios, and the detection results were then analyzed to verify the realistic performance of the different methods. The final detection results are shown in Figure 6.

From the description of the experimental results in Figure 6, it can be concluded that UFSLD has limitations in accurately detecting the belt edge line under inadequate lighting conditions and in regions where the features were not obvious. In Scene 2, although the light conditions were good and UFSLD used a second-order difference constraint, the extracted belt edges appeared as irregular curves, causing deviations in the position of some edge points and leading to false alarms, which affected production. Despite similar metrics to RBDNet in Table 5, the practical extraction results of UFSLD were significantly different and did not meet the requirements for practical application in coal belt scenarios.

In contrast, the L-CNN algorithm performed well in various scenarios except for coal mines. In the coal mine scenarios, L-CNN struggled to discriminate straight lines due to the complex environment, lack of light, dust, and other unusual factors. The method relied on identifying straight line segment endpoints, which were difficult to determine accurately in coal mines. While the extraction effect was better for other facilities within the scene, the detection performance for the belt edge line was not satisfactory in terms of accuracy and practical requirements.

These observations highlight the limitations and challenges faced by UFSLD and L-CNN in detecting belt edge lines in coal mine scenarios, indicating the need for alternative methods, such as the proposed RBDNet, to address these issues effectively.

### 3.4. Belt Edge Line Detection under Abnormal Conditions

**Figure 7:**
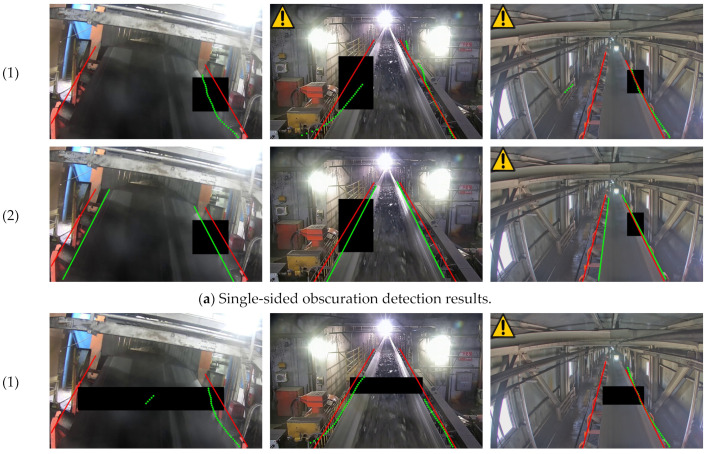

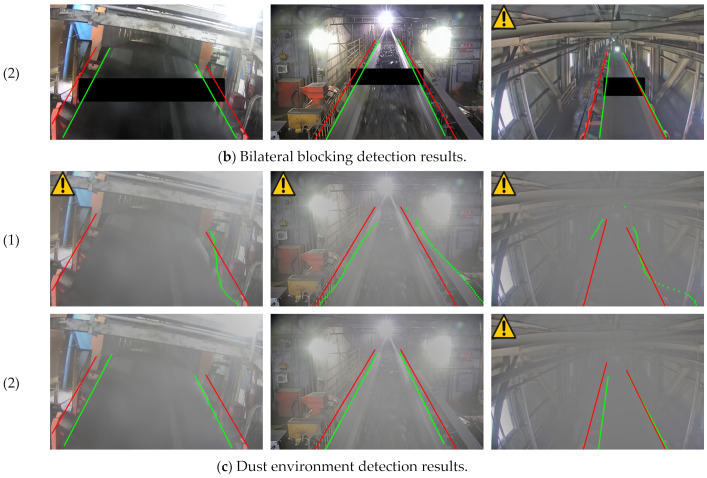
Abnormal state belt misalignment detection result map. Figure 7 (1) is the detection result for UFSLD and (2) for RBDNet.

In application scenarios such as coal transport belts, there is inevitably the influence of factors such as dust and large foreign objects obscuring the belt edge, resulting in weakened belt edge information. In addition, staff may work in the vicinity of the coal transport belt, and shadows caused by light can lead to reduced or even indistinguishable contrast at the belt edges in this area. Therefore, in this paper, the detection effect under shading conditions was verified for possible shading and dust phenomena in the belt operation. The UFSLD algorithm in Section 3.2.3, which has a relatively good detection effect, was used to carry out the comparison experiments with two different forms of black areas instead of the shading of the belt edge by foreign objects of different sizes and the shading of the belt edge by the limbs or shadows of people. The detection effect is shown in Figure 7.

In this paper, belt edge line detection in the scenarios with area occlusion and heavy dust is discussed. In the case of area occlusion, the detection result was slightly affected, resulting in a deviation in the belt edge line from the actual position. However, by utilizing contextual information, a priori knowledge obtained during training, and unobscured edge lines, the algorithm can generate belt edge lines by constraining the features of the remaining belt edge lines. This helps to mitigate the impact of area occlusion on the overall detection performance.

Regarding heavy dust, the method proposed in the paper effectively overcame its effects on belt edge detection. Dust did not significantly impair the detection results.

The deviations in the belt edge line were primarily attributed to the prediction of the edge information in the single-sided and double-sided masking regions based on the contextual and location information. The deviation occurred due to candidate point displacement and subsequently affected the fitted belt edge line. The double-sided masking area generally exhibited slightly larger deviations compared to the single-sided masking area.

In summary, the proposed method addresses the challenges posed by area occlusion and heavy dust in belt edge detection, providing more accurate results despite slight deviations in the edge line under certain conditions.

## 4. Discussion

First, this paper provides a further analysis and comparisons to evaluate the effectiveness of the RBDNet method. The key findings are described as follows:

(1) Lighter Model

The experiments conclude that using ResNet18 as the backbone network results in a slightly lower accuracy compared to ResNet50 but with a significantly faster processing speed. This tradeoff is suitable for belt deviation detection scenarios that have stricter requirements in both accuracy and speed. The video stream detection speed is increased by 2 fps, 14 fps, 32 fps, and 35 fps compared to the other algorithms.

(2) Improved Accuracy

Compared to the other algorithms such as UFSLD, L-CNN, LSD, and Hough transform, the RBDNet method demonstrates a higher accuracy in belt edge line detection. This paper reports an improvement of 0.4%, 15.9%, 45%, and 78.8% in belt edge line detection accuracy compared to these algorithms. Similarly, the F1-measure also improve by 0.3%, 9.9%, 32.6%, and 72%, respectively. Verification using video stream data from coal mine conveyor belt operations confirms the requirements in terms of accuracy and speed.

(3) Scene Adaptability

The RBDNet method exhibits better adaptability to various scene conditions compared to other algorithms. It can handle poor lighting conditions, dust, and occlusion by foreign objects. Poor illumination and dust minimally impact the detection results, while regional occlusion may slightly affect the performance. The algorithm can extract belt edges effectively throughout different times of the day, overcoming variations in illumination. The experiments with area masking also show positive results without compromising speed or accuracy. In contrast, algorithms such as UFSLD are less effective in detecting belt edges under abnormal conditions, further validating the superiority of the proposed algorithm and its improvement strategy.

These findings highlight the advantages of the RBDNet method, including its balance between accuracy and speed, superior performance compared to other algorithms, and robustness in handling adverse conditions.

## 5. Conclusions

Aiming at the existing belt deviation methods that cannot be practically applied in industry, this paper proposes a real-time belt deviation detection method based on the depth edge feature and gradient constraint (REDNet). The feature extraction is carried out with ResNet18 as the backbone network, the global feature analysis is carried out through the softmax classifier in the fully connected layer, the prediction of the model is achieved by using the first-order difference constrained belt edge, the accurate belt edge straight line is obtained by linear fitting through least squares and compared with the threshold value, and the real-time runaway detection of the belt is realised. Based on the monitoring video actually collected on site, the industrial belt deviation detection dataset was established. By comparing and analyzing the results of various experiments, the following conclusions are drawn:

(1) In the different sizes of the ResNet network models, ResNet18 guarantees the detection accuracy under the premise of improving the detection speed. The detection reaches 41 frames/s, and the detection accuracy is 96.21 percent.
(2) The belt edge position constraints based on the feature classification, edge attention mechanism with first-order differential loss can effectively improve the accuracy of detection, and the accuracy rate can reach 96.21 percent.
(3) Compared with UFSLD, L-CNN, LSD, and the Hough transform, the accuracy of the proposed REDNet is improved by 0.4%, 13.9%, 45.9%, and 78.8%, and the F1 scoring index is improved by 0.3%, 10.2%, 32.6%, and 72%, respectively, and the accuracy, precision, and speed of RBDNet are optimal.
(4) The proposed RBDNet is well adapted to scenarios such as insufficient light, occlusion of the belt area, and excessive dust in the detection area, and accurate detection results can be obtained.

The proposed method has been practically applied in REDNet with good results. In the future, we will build a more lightweight model to achieve edge computing at the camera end near the belt to reduce the requirement of hardware performance and improve the applicability of the model.

## Figures and Tables

**Table 1:** Training Parameters.

Training Metrics	Parameters	Training Metrics	Parameters
batch_size	16	Hyperparameter	0.9
Epochs	300	Optimizer	Adam
Weight decay	0.0001	Learning rate	0.0004

**Table 2:** Experimental software and hardware configurations.

Name	Configuration
Operating system	Windows 10 Professional
Experimental framework	PyTorch1.5.0 [35]/Python3.6.13
CPU	11th Gen Intel(R) Core(TM) i7-11700
GPU	NVIDIA Titan Xp(12G)
RAM	16G

**Table 3:** Performance statistics of different backbones.

Backbone	Accuracy	F1-Measure	Frame Rate
ResNet18	96.21	90.5	41
ResNet34	96.27	90.7	21
ResNet50	96.31	90.8	15

**Table 4:** Ablation experiments.

Baseline	Feature-Based Belt Edge Position Prediction	Transformer	First-Order Differential Structural Loss	Accuracy	F1-Measure
√				92.58	87.06
	√			95.36 (+2.78)	89.23 (+2.17)
		√		93.97 (+1.39)	88.36 (+2.30)
			√	94.24 (+1.66)	88.97 (+1.93)
	√	√		95.77 (+3.19)	89.56 (+2.50)
	√		√	95.89 (+3.41)	89.48 (+2.42)
	√	√	√	96.21 (+3.63)	90.5 (+3.63)

**Table 5:** Performance index table of different algorithms.

Algorithms	Ours	UFSLD	L-CNN	LSD	Hough
Accuracy	96.2	95.8	83.0	52.0	20.4
F1-measure	90.5	90.2	81.2	61.0	25.3
Frame Rate	41	39	27	9	6

## Data Availability

The data that support the findings of this study are available on request from the corresponding author, [X.X], upon reasonable request.
